# The Role of Trust in COVID-19 Vaccine Acceptance: Considerations from a Systematic Review

**DOI:** 10.3390/ijerph20010665

**Published:** 2022-12-30

**Authors:** Alessandro Sapienza, Rino Falcone

**Affiliations:** Institute of Cognitive Sciences and Technologies, National Research Council of Italy (ISTC-CNR), 00185 Rome, Italy

**Keywords:** COVID-19, vaccine hesitancy, vaccine acceptance, trust, confidence, trust in vaccine, trust in government

## Abstract

The goal of this research was to provide an overview of the role of trust in determining COVID-19 vaccine acceptance. Trust proved to be a key issue in all the strategic phases of the pandemic, a decisive element for the success of the worldwide vaccination campaign. By introducing a comprehensive systematic review of the state-of-the-art (*N* = 43), we intend to shed light on the various forms of trust that have been considered and how these relate to citizens’ vaccine acceptance. The analysis shows that trust has been used extensively, with particular reference to the COVID-19 vaccine, governments, manufacturers, healthcare systems, and science. A more in-depth analysis has also allowed us to evaluate the role that these factors have had and the social phenomena in which they have been decisive. Most notably, we proved that, in the different contributions, trust in the COVID-19 vaccine has a strong correlation with vaccine acceptance (R = 0.78, *p* < 0.01). Overall, vaccine acceptance emerges as a complex phenomenon that needs to be understood through the strictly interlaced relations of trust in the various factors coming into play. Besides clarifying what happened in previous years, the considerations included in this work also represent an important and useful interpretative framework to help public institutions and the healthcare system in the future.

## 1. Introduction

The COVID-19 pandemic represented and still represents one of the greatest challenges that our society has had to face. Although lockdowns and restrictions appear to be a thing of the past, and despite the realization and distribution of effective vaccines, the COVID-19 pandemic persists with resurgent waves. Different strategies and solutions have been proposed to handle all of the unprecedented problems that have arisen, both at the beginning of the pandemic and then afterwards.

Indeed, resilience of countries to COVID-19 seems to be linked to a common key element: trust [1,2]. Most notably, trust in the governments has had a fundamental role in determining the acceptance of the drastic restrictive measures introduced [3,4]. As Bollyky ([5], p. 1) reports, “when confronted with a novel virus for which there is no pre-existing treatment or vaccine, the most effective way for a government to protect its citizens is by convincing them to take measures to protect themselves and one another”. This is why trust made the difference in tackling the pandemic, especially for democracies.

Besides trust in governments, even trust in the healthcare system has proven to be decisive. Antinyan [6] underlines that the low trust citizens exhibit toward healthcare institutions can induce them to get engaged in a number of uncooperative behaviors, which can severely undermine the efforts that governments exert to stop COVID-19, especially in low- and middle-income countries. Along the same line, Alijanzadeh [7] found that trust in the healthcare system mediated the association between an individual’s risk perception and performing preventive COVID-19 behaviors.

Last but not least, trust has played a key role in the advancement of mass vaccinations [8,9]. Studies regarding COVID-19 vaccine acceptance have been published since the early phases of the pandemic [10,11,12,13,14], showing great variations in research questions and results. As we will show below, in our analysis, trust has emerged as a pivotal factor in many of these studies.

Nevertheless, despite its importance, a detailed analysis of how this dimension has been considered within the different works is still lacking. A comprehensive understanding of how the different forms of trust have been characterized and to what extent they have influenced citizens’ decisions is fundamental to overcome this research gap.

After more than two and a half years, we can draw conclusions from what happened and what social phenomena have emerged: a complicated relationship with government [15] and health institutions [16,17], the role of information and communication in general [18], the spread of fake news and scientific disinformation [19,20], etc. Specifically, within this work we were interested in investigating the role of trust, in its different forms, in determining vaccine acceptance. Several reviews dealing with attitudes and hesitancy toward COVID-19 vaccination have been published [21,22,23,24,25,26]. However, to the best of our knowledge, none of them has specifically focused on trust as one of the main determinants of vaccine acceptance.

Thus, the objective of this review is the identification of the various aspects of trust considered in relation to vaccine acceptance and their salience within the current literature.

## 2. Methods

### 2.1. Design

The realization of this review has been inspired by the following research questions: (1) what forms of trust were considered in the different studies; (2) what role does trust play in determining their acceptance by individuals; (3) which socio-demographic, mental and behavioral factors influence trust in these vaccines.

### 2.2. Search Strategy

The research was conducted in October 2022, by making use of PubMed and Google Scholar. Specifically, we used combinations of the following keywords: ‘’COVID-19”, “coronavirus”, “vaccine”, “survey”, “hesitancy”, ‘’acceptance”, ‘’trust”, and ”confidence”. After a first phase concerning contributions search, a total of 171 peer-reviewed contributions was retrieved, 25 of which were excluded as duplicates. Thus, title and abstract screening were conducted for the remaining 146 articles, 9 of which were excluded. The selected articles were further reduced according to the following inclusion criteria:

1. *Value*, the article must address the problem of subjects’ COVID-19 vaccine acceptance/hesitancy, together with its determinants;
2. *Inclusion of trust*, the work should at least consider one type/dimension of trust as a determinant of COVID-19 vaccine acceptance;
3. *Relevance*, the contribution must be written in English and published in a relevant journal. We considered 2.5 as a cut-off value for the impact factor;
4. *Accessibility*, the full article should be accessible via one of the previously mentioned portals.

**Figure 1:**
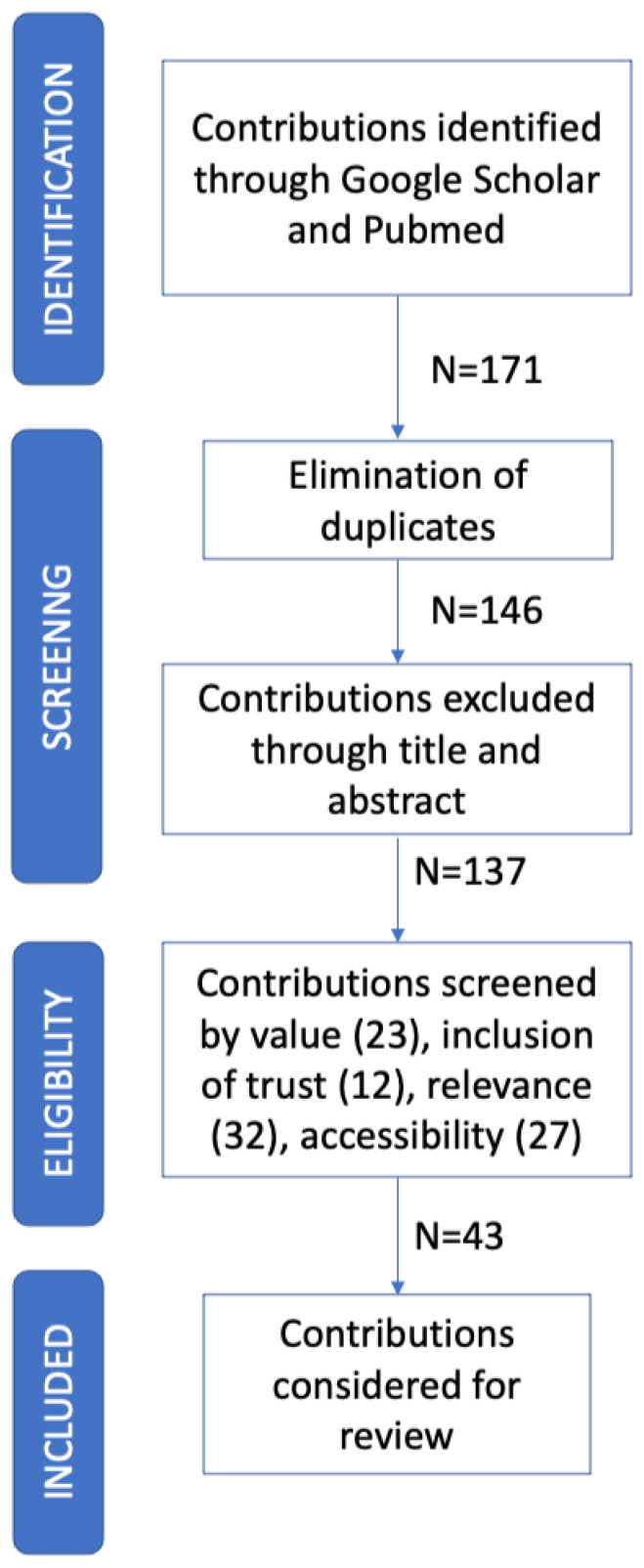
Flow diagram of contribution retrieval and selection.

Overall, a total of 43 papers has been considered (see Figure 1).

## 3. Results

### 3.1. Data Extraction

The details extracted from the contributions included country, surveying period, sample size, key questions, vaccine acceptance, use of trust, and relevant factors and their effects on vaccine acceptance and/or on trust in the vaccine.

The studies considered are very heterogeneous. Table 1 summarizes their main characteristics. In almost all cases, data on subjects were collected through surveys (cross-sectional *N* = 39 and longitudinal *N* = 3), but one study collected data through social networks (*N* = 1). The period of analysis ranges from March 2020, at the very beginning of the pandemic, up to the most recent data concerning January 2022. Most of the studies were conducted in the USA (*N* = 14) and several of them consider multiple countries (*N* = 7). As for the sample size, it varies from a minimum of 60 to a maximum of 525,809 subjects (mean *N* = 26,279, median *N* = 2126).

Findings were synthesized in a narrative way and presented through the use of summary tables and graphs, in order to illustrate in a structured way the role of the different dimensions.

These studies examined a range of trust dimensions in relation to vaccination, starting from trust in the COVID-19 vaccine, in the government, healthcare system, and so on. Table 2 summarizes the various instances of trust that have been characterized as predictors of vaccine acceptance in the considered contributions.

The most widely considered dimensions concern vaccines’ safety (*N* = 20) and efficacy (*N* = 17). Concretely, although these are not direct forms of trust, safety and efficacy are fundamental components of trust in general and, in the specific case, of trust in the COVID-19 vaccine. Remarkably, even the SAGE (Strategic Advisory Group of Experts on Immunization) working group identifies them, in the 3C and 5C scales, as key elements for assessing vaccine trust [70]. Safety is based on the belief and expectation that one will not be harmed by the addressee of one’s trust (in this case, the vaccine) [71]. Indeed, this is a component of trust: I do not expect to receive damage from you. In the specific case in question, safety is linked to the absence of short- and long-term side effects. As far as efficacy is concerned, this aspect belongs to the dimension of competence, a necessary feature for a positive evaluation of the addressee’s trustworthiness. In the specific case, efficacy concerns the belief and the expectation that the vaccine is actually able to protect individuals from COVID-19.

After safety and efficacy, other dimensions of particular importance are trust in governments (*N* = 16) and trust in the COVID-19 vaccine (*N* = 15). For the sake of completeness, we report that some papers considered trust in the COVID-19 vaccine, but they did not analyze its role in vaccine acceptance. Thus, the total number of works considering trust in the COVID-19 vaccine is even greater (*N* = 19).

Other types of trust taken into consideration concern the health system (*N* = 9), sciences (*N* = 7), manufacturers (*N* = 4), vaccines in general (*N* = 4), religion (*N* = 1), social trust (*N* = 1), and commercial profiteering (*N* = 1).

In Table 2, “positively related” means that there is a positive relationship between the element considered and vaccine acceptance. By “negatively related” we mean the opposite relationship: vaccine acceptance decreases as the variable considered increases. To give a clarifying example of a positively related variable, the higher the trust in the COVID-19 vaccine, the higher is vaccine acceptance. The lower the trust in the COVID-19 vaccine, the lower is vaccine acceptance. Conversely, the negative effect of concerns about commercial profiteering indicates that its increase will result in a decrease in vaccine acceptance, while its reduction determines an increase in vaccine acceptance. It is worth noting that almost all of these dimensions are positively related to vaccine acceptance. We encounter only three exceptions to that:

1. Concerns about commercial profiteering: the negative relationship, identified by Gerretsen [35], is easy to understand, since believing that the production and distribution of vaccines are motivated and conditioned by profit reasons leads reasonably to believe that other interests may prevail, not necessarily linked to the protection of health.
2. Trust in religion: Rozek [57], in their seventeen-country study, reported that trusting religious leaders increases vaccine hesitancy. In support of this, Jafar [39] noted that Muslim respondents were found to be less confident towards vaccines compared to non-Muslim respondents. However, Mueangpoon [50] found that religion has a non-significant (n.s.) effect on vaccine hesitancy, while Riad [56] reported that the vast majority of participants (87.4%) stated that their religious values did not impact their vaccination decision.
3. Trust in government: while trust in the government is always detected as positive, Trent [65] identified an opposite situation in the USA. We will elaborate on this point further, in the specific subsection related to trust in the government (see Section 3.3).

### 3.2. Trust in the COVID-19 Vaccine

Trust in the COVID-19 vaccines is perhaps the most pivotal element in influencing vaccine acceptance. This is demonstrated by the large number of works that took it into consideration, but also by the fact that the World Health Organization (WHO) itself has identified it as a strategic element, both for vaccines in general [72] and for the specific COVID-19 vaccine [73]. However, in the different contributions, there is no agreement on how this dimension should be assessed:

1. 3C/5C scale [70]: Abdou [27], Dorman [32], Liu [47], Machida [48], Parsons [53];
2. Vaccine Confidence Index [74]: Han [37];
3. Personalized scale: Daly [31], Gerretsen [35], Hou [38], Jafar [39], King [42], Mueangpoon [50], Wang [67], Williams [68];
4. Single item/directly assessed: Falcone [33], Latkin [45], Soares [61];
5. Not defined: Jennings [40], Trent [65].

Among these works, only six also investigated the predictors of trust. Most of these works focused on socio-demographic variables, while only a few considered behavioral, cognitive, and beliefs factors. Among the socio-demographic variables that influence trust in the COVID-19 vaccine, the following dimensions stand out:

1. Gender: men are generally more inclined to trust than women [27,45,67,68];
2. Age: Wang [67] and Williams [68] identified a positive correlation with trust in the vaccine, while for Latkin [45] it was n.s.;
3. Educational level: the effect of this variable is somewhat controversial, as some papers found that more educated people have more trust in the COVID-19 vaccine [33,39], while others found the opposite relation [27,67] and some stated that it is n.s. [45,68];
4. Income: this dimension was found to either be positively related to trust [33,68] or n.s. [45].

Other relevant socio-demographic indicators include: religion [39]; previous history of COVID-19 [27]; taking annual flu vaccine [27]; following COVID-19 protective measures [27]; vaccine cost [27]; financial consequence of the pandemic [33]; race [45]; political party [45]; being in a vaccine priority group [67]; region of residence [68].

As far as it concerns behavioral, cognitive, and beliefs factors, the following dimensions have been found to have a positive impact on trust: vaccine safety; vaccine efficacy; trust in manufacturer; trust in regulators; trust in government; believing that not much can be done to prevent getting the coronavirus; trust in social norms. Conversely, the following dimensions have a negative effect on trust: concerns for safety; distrust in vaccines in general; general skepticism; profit distrust; doubt in efficacy.

**Figure 2:**
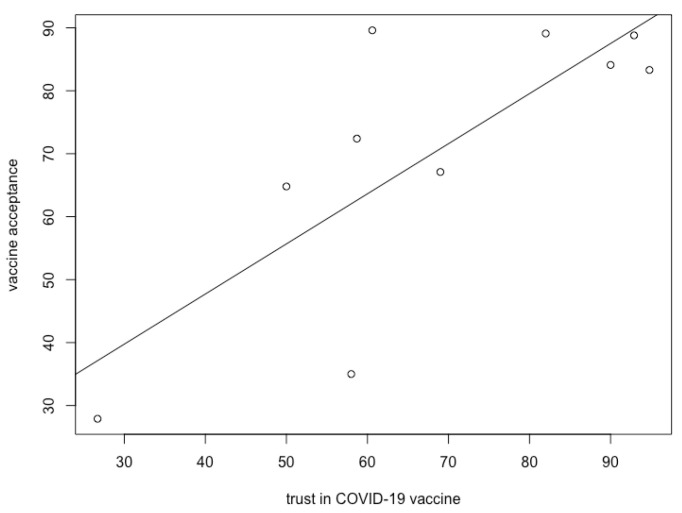
Regression chart on the relationship between trust in the COVID-19 vaccine and vaccine acceptance.

After the initial analysis of the characterization of trust in the COVID-19 vaccine, we investigated the relationship between this dimension and vaccine acceptance. Among the considered works, only 10 reported the values of both these dimensions. We report these values in Table 3. More specifically, these represent the average values of subjects’ vaccine acceptance and trust in the COVID-19 vaccine, as reported by the various studies. To standardize and compare these values on a single scale, we have chosen to report them as a percentage. The most remarkable result about trust in the COVID-19 vaccine is that it strongly correlates (R = 0.78, *p* < 0.01) with vaccine acceptance, (see Figure 2). Indeed, such a tight correlation sheds light on the strong role of this type of trust in determining vaccine acceptance. This result assumes particular relevance, if we think that trust has been assessed using different approaches, considering different time periods and in several different countries.

Analyzing Table 3 in greater detail, the case of Mueangpoon [50] stands out, having a 29% difference between acceptance and trust. Contextual information may provide a plausible explanation for this. As the author reports, initially, the government relied solely on the two vaccines Sinovac and AstraZeneca, which were thought to have low efficacy. However, the intention to get vaccinated was high, so that citizens turned to vaccines offered by private companies, despite having to pay for that.

Another interesting case is that of Soares [61]. Even here, the difference between those who trust and those who choose to get vaccinated is high. Actually, the authors reported that only 9% of the subjects did not intend to get vaccinated, while 56%—a significantly high part of the sample—would wait before taking the vaccine. The positions of the latter are fundamentally different from those who oppose vaccination. The subjects not intending to get vaccinated have in fact clear-cut and well defined positions. Similarly to the former, these are not confident in the health service, and believe that information provided by health authorities is inconsistent and contradictory and that the measures put in place were insufficient. However, these aspects are very pronounced in their case. Furthermore, they believe that the risk of getting COVID-19 infection is minimal, as well as that of developing complications. On the contrary, by analyzing the subjects who prefer to wait, a substantial feeling of uncertainty emerges. The authors associate this uncertainty with the perception of safety and efficacy, stating that the government should implement an information campaign on such a topic, in order to increase trust in vaccines. To conclude, this contribution highlights another important phenomenon that occurred: the climate of substantial uncertainty emerged in the first phases of the vaccination campaign, which prompted subjects to postpone any decision about vaccination.

### 3.3. Trust in Government

Although this dimension has extensively been taken into consideration in the works, even more than trust in COVID-19 vaccines, it also appears to have a controversial nature. In fact, although the object of trust is always the government, the purpose of the trust taken into consideration is not always the same. To clarify this point, let us examine how the different authors have characterized it:

1. Brindle [30], Falcone [33], Gerretsen [35], Jennings [40], Park [52], and Strupat [63] considered the government’s ability to manage the pandemic;
2. Wang [67] considered trust in the government for communicating information about the COVID-19 vaccine;
3. Mueangpoon [50] estimated trust in the government about encouraging COVID-19 vaccination;
4. Szilagyi [64] considered the purpose of ensuring that the COVID-19 vaccine is safe for the public;
5. Rozek [57], Allington [28], and Jennings [40] did not specify the purpose of the trust, considering a general form of trust in government;
6. Trent [65] introduced a scale that considers generic trust, pandemic management, and trust as a source of information about the pandemic;
7. Goodwin [36] used a scale that considers generic trust, pandemic management, and vaccination management.

From the works we considered, a positive relationship almost always emerges between trust in the government and the decision to get vaccinated. However, there are some cases that deviate from this trend and that are interesting to analyze in more detail.

As a first interesting case, Schernhammer [60] (Austria, November–December 2020) found that hesitation increases for those who voted for the opposition parties and this effect was even stronger for those who did not vote at all, compared to those who instead voted for the governing parties. At the time of the survey, there was a right-wing popular government, which was basically in favor of vaccination. As far as it concerns the opposition parties, we find a multi-faceted situation. The Social Democratic Party of Austria and The New Austria and Liberal Forum were broadly pro-vaccination [75,76], while the Freedom Party of Austria took more distant positions [77]. At the same time, in that historical period, the People Freedom Fundamental Rights group gathered electoral support precisely because of its marked no-vax connotations [78]. Therefore, it seems appropriate to state that the greater hesitation of those who did not recognize themselves in the government parties can only in part be traced back to the positions of the opposition parties, while it appears more as an effect of distrust towards the constituted authority, as also confirmed by the greater effect size in those who abstained from voting.

Furthermore, Brindle [30] found a substantially low trust in the UK government. The author reported that trust in the quality of government decision making changed substantially between April 2020, when 52.7% of respondents said that the government was making good decisions, and December 2020 (21.7%). In addition, Trent [65] noteed that, between July and September 2020, trust in the UK government was n.s. in predicting vaccine acceptance. These results should be interpreted in light of some background information.

The first point concerns the sharp change of course in the management of the pandemic. Prime Minister Johnson initially played down the threat posed by the pandemic [79]. However, immediately after his contraction of the virus, the tone quickly changed: in March 2020 a full lockdown was instituted. Therefore, the initial high public support declined as the government was forced to make several strategy changes. In a short time, the UK had the largest number of victims in Europe [80].

The second key event in the UK concerns the so-called Dominic Cummings scandal, which emerged in late May 2020. Dominic Cummings was a senior adviser to the Prime Minister, who breached lockdown rules, traveling more than 400 km to a family estate with his child and wife who had COVID-19 symptoms [81]. Cummings never apologized for his actions, and was largely supported by the UK government.

Another situation of particular interest concerns a negative correlation in the USA in the period of July–September 2020. Trent [65] reported that participants with high trust in their current government were less likely to be willing to receive the vaccine. Even in this case, these results must be interpreted in light of the political situation in the time frame taken into consideration.

Since the outbreak of the coronavirus, the USA President Donald Trump had been downplaying the risks of COVID-19—questioning the effectiveness of masks, touting unproven treatments, and criticizing his own health experts, including Dr. Anthony Fauci, director of the National Institute of Allergy and Infectious Diseases [82]. Trump asked to slow down on testing since ‘’when you do testing to that extent, you’re going to find more people, you’re going to find more cases”. On the other hand, Fauci asked to carry out more testing [83]. Trump has often taken unscientific positions. For instance, he has defended the use of hydroxychloroquine to ward off coronavirus, contradicting his own public health officials [84]. Lastly, the Trump administration promoted a hyper-ambitious plan to develop and manufacture hundreds of millions of COVID-19 vaccine doses by the end of 2020 (‘’Operation Warp Speed”) [85]. Trump stated that a coronavirus vaccine would probably have been available in October 2020, contradicting the top health officials who said it would have been very unlikely [86].

### 3.4. Trust in Manufacturers

Trust in Manufacturers has been clearly identified as a component positively related to vaccine acceptance.

Latkin [45] reported that, in the United States, pharmaceutical companies are the most poorly regarded industry [87]. This well-documented perception is likely to have led to mistrust in pharmaceutical companies’ ability to distribute safe and effective COVID-19 vaccines.

Falcone [33] simultaneously investigated trust in regulators (public institutions and drug safety authorities; 77.5%) and manufacturers (83.5%). This shows that the main motivation behind this trust difference is that regulators are trusted more for their intentions (86.6%) than for their competence (73.8%), while the opposite is true for manufacturers (trust in competence = 91.8%, trust in intentionality = 76.6%). In addition, through structural equation modeling, Falcone showed that only trust in manufacturers is crucial in generating trust in vaccines. According to this, the specific role of regulators is to be impartial evaluators of pharmaceutical products, thus improving trust in manufacturers, which in turn boosts trust in vaccines.

Szilagyi [64] noted that while trust in the development processes added substantial predictive value when combined with each of the other trust domains they considered (generalized trust, trust in vaccine efficacy and safety, trust in healthcare providers, trust in sources of information), the predictive value of trust in the development processes itself did not appreciably improve when combined with the other trust domains. Thus, the author’s conclusion is that trust in the approval/development processes explains virtually all of the covariation between the other trust domains and stated vaccination likelihood.

### 3.5. Trust in Healthcare System and Science

Both of these dimensions positively relate to vaccine acceptance. Although they are very close to each other and often analyzed together, they are generally evaluated from different points of view.

As far as the healthcare system is concerned, it is mainly considered in its role of informant. Most notably, less trust in the health system makes citizens more sensitive to misinformation about the COVID-19 vaccine [67]. In this connection, a further aspect that emerged concerns the inconsistency and contradictory aspects of the information reported by the health authorities [61].

Latkin ([45], p. 6) suggested that “to address low rates of trust in the COVID-19 vaccine, vaccination promotion efforts should both involve and be informed by health professionals, including physicians, nurses, pharmacists, community health workers, and mental health therapists, who have ongoing relationships with patients and likely have increased capacity to build trust”. Szilagyi [64] reports that subjects intending to get the vaccine have higher trust in information coming from healthcare providers, the CDC, the WHO, and local public health officials. Therefore, the author suggests that public health and vaccine leaders should work with news organizations on effective messaging about the effectiveness and safety of the vaccine.

Lastly, Stoler [62], analyzing the history of distrust of Black Americans in the healthcare system, drew attention to this lack of trust, stating that it must not be confused with other phenomena due to demographics or conspiracy thinking. As such, it requires the medical establishment to demonstrate its trustworthiness in order to begin to mitigate vaccine hesitancy.

On the other side, Science is mainly taken into consideration in its supervisory role, both as regards the scientific foundation of available COVID-19 vaccines [34], but also for the legitimacy of the decisions and actions taken by governments. In general, greater trust in science corresponds to greater vaccination intention [30,57,66].

Remarkably, Jennings [40] stated that vaccine-willing participants were more likely to see the government as having followed the science.

Allington [28] found that the trust in scientists working in universities and for companies both have a positive effect on vaccine acceptance, but the former has a greater weight than the latter.

Latkin [45] stated that societal trust in science may have further eroded throughout the fragmented response to the COVID-19 pandemic in the United States. In support of this claim, the author reported that a Pew Research poll conducted in late April 2020 found that public trust in science is low, with only 52% of Democrats and 27% of Republicans reporting confidence in their belief that scientists act in the best interest of the public [88].

## 4. Discussion

In recent years, we have witnessed a very critical moment for our society. The arrival of COVID-19 has brought about enormous changes [89] and has generated situations of great social contrast. On the one hand, citizens have had to rely on institutions to determine adequate solutions to these new problems. On the other hand, the implementation of these solutions depended on strong social cohesion, which was nevertheless severely tested by the required sacrifices. In this double and close bond of entrustment of citizens to institutions and vice versa, a long and challenging path has been traveled, in which trust has played a leading role. Specifically, in this work we focused on the issue of vaccine acceptance. To the best of our knowledge, this is the first study reviewing how the different forms of trust affected vaccine acceptance.

Indeed, the phenomenon of vaccine hesitancy [90,91] has been strongly studied in the past and, in particular, in recent years. With reference to the COVID-19 vaccine, this issue has led to strong social problems: vaccine production; ethical and fair distribution among the various nations and access priorities among the various social classes [92]; right to vaccination [93]; paid vaccination; mandatory vaccination [94]. The various nations, considering their specific reference context, have adopted different strategies to foster vaccine acceptance, obtaining results to a greater or lesser extent close to what expected.

Given the critical nature of this situation, investigating what fostered vaccine acceptance becomes pivotal. Understanding citizens’ psychological reactions could strongly help the authorities realize better intervention policies and communication strategies in the future [95]. Remarkably, it has been shown that, during pandemics, citizens’ trust in public institutions has a key role [96] in determining their willingness to adopt recommended behavior [97]. Indeed, our analysis on trust in the government has provided useful insights in this regard, highlighting a sometimes conflicting relationship between those who define the guidelines and those who must follow them. In general, our data confirm the positive relationship identified in the literature even for vaccine acceptance. However, what emerges is a very complex picture, given the specific characterization of the different nations. In fact, the actions and decisions of the government, if perceived as inappropriate or discordant, may influence the citizens’ perception to such an extent that the trust in the government can become even n.s. or negatively related to vaccine acceptance.

Another precursor worth mentioning emerged in our analysis is trust in the COVID-19 vaccine. As evidence of its pivotal role, we were able to quantify a strong correlation with vaccine acceptance (R = 0.78, *p* < 0.01). Despite the presence of such a tight bond, a more in depth analysis has shown us how other factors may intervene in this relationship. A noteworthy example is the uncertainty that has characterized this vaccine, specifically concerning the aspects of safety and efficacy. A particular note should be made about the dimension of safety. Indeed, it is worth emphasizing that this is linked both to short-term and long-term side effects. Although these are treated in a condensed manner in a single variable, there are significant and relevant differences between these two categories. While information was presented on short-term side effects, the need to face the impelling threat made it impossible to wait for any evaluation of longer-term side effects. Unfortunately, it was not possible to evaluate the impact of these two aspects separately. However, it would have been important to understand whether the concern about side-effects was more related to short-term or long-term effects, also by evaluating their evolution over time at different points in the distribution.

In this regard, notwithstanding the strong information campaign to reassure citizens, several no-vax movements have sprung up [98,99,100], fed by disinformation and fake news spreading on the web and social networks. Yet trust is itself the proper tool to address these risks. First of all, trust is instrumentally necessary for the functioning of our society [101,102,103]. Trust is the construct behind every social interaction [104,105]. Basically, we cannot think of being able to evaluate the efficacy and safety of the vaccine ourselves: we must rely on those who have these skills and who are in charge of this task. In practice, this requires a recognition by the citizens of the trustworthiness of the processes of selection and evaluation of the skills that the society prepares. Second, risk and uncertainty are the fundamental analytic presuppositions of trust [71,103], or rather the elements that describe the situations where trust is important for predictive purposes. Trust presupposes the subjective propensity of the truster to accept a given degree of uncertainty and ignorance, and a given perceived amount of risk. Therefore, trust is the proper tool that should be used to tackle the problem of uncertainty. It is precisely on this dimension that we must work to encourage citizens to cooperate, adopting the required behaviors.

With respect to manufacturers, we found a critical issue regarding the dimension of intentionality, which in turn is closely linked to citizens’ concerns about commercial profiteering. A mixture of conspiracy and fake news has fueled doubts about vaccines, as assisted by an extraordinarily fast vaccine development processes [106], much shorter than is normally the case. Although various explanations have been provided in this regard, demonstrating for example that it was possible to drastically shorten the timing also thanks to the immediate allocation of large funds by world governments, the doubt about the transparency of the manufacturers’ motivations has nevertheless remained a strong element of skepticism. In this connection, the role of two “super partes” institutions, the Healthcare System and Science, was also fundamental to provide clear information and to legitimate (or not) the choices made by governments. Indeed, the results of this review underline the importance of reliable information on vaccines and that public health and vaccine leaders should work with news organizations on effective messaging about the effectiveness and safety of the vaccine. However, while this plays a critical role, it may not be an easy task. Even if these primary sources provide neutral information, the specific editorial lines of the media could somehow alter or subvert the neutral message that is provided to the general public [107,108,109], such that the ‘’objective” reporting of information on vaccines is countered by a non-neutral editorial process.

## 5. Conclusions

Overall, the results of this review clearly highlight how important trust was in determining the adherence to vaccination campaigns that took place worldwide. However, as a limitation, this review found that most of the contributions lack a structured approach to trust. Once having identified the various types of trust that affect vaccine acceptance, it is also important to understand if and how they relate to each other. On the other hand, although it is useful to quantify trust and its effect as a predictor, it is also fundamental to investigate its reasons: what aspects influence it and what beliefs of individuals determine it. Only in this way it is possible not only to reach an in-depth understanding of the behavioral and social complexity that has emerged during this crisis, but also to identify how it is possible to intervene to obtain or improve community adherence to the required measures.

This study is not without limitations. Most notably, it is important to underline that, although we identified many phenomena and trends, it would be improper to consider them as consolidated because of the limits in the different contributions analyzed. Among the various limits, it must be considered that the same concepts have been defined in different ways (or not defined at all) within the studies. In addition, it is not always easy to consider the effects and phenomena that occur in specific studies without rich contextual information related to the historical moment of data collection. Therefore, our results can be considered as an important meta-analysis, to be used as a starting point for future more in-depth analyses. In this connection, despite its critical role, it should be emphasized that trust has not been sufficiently studied in these contexts. This implies that there is not yet enough data to carry out more complex and articulated analyses.

## Figures and Tables

**Table 1:** Characteristics of the sample.

Contribution	Country	Surveying Period	Sample Size
Abdou [27]	13 Arab countries	Dec 2020–Feb 2021	4474
Allington [28]	UK	Nov–Dec 2020	4343
Babicki [29]	Poland	Dec 2020–Mar 2021	2022
Brindle [30]	UK	Dec 2020	4535
Daly [31]	USA	Oct 2020–Mar 2021	7420
Dorman [32]	USA	Oct–Nov 2020	26,324
Falcone [33]	Italy	Mar–Apr 2021	4096
Fernández [34]	USA	Jan–May 2021	1068
Gerretsen [35]	USA and Canada	May 2020, Jul 2020, and Mar 2021	7678
Goodwin [36]	Israel, Japan, and Hungary	Jan-Apr 2021	2127
Han [37]	China	Nov 2020	2126
Hou [38]	USA, UK, Brazil, India, China	Jun–Jul 2020	12,886
Jafar [39]	Malaysia	Mar–Apr 2021	1024
Jennings [40]	UK	Dec 2020	1476
Kerekes [41]	US, China, Taiwan, Malaysia, Indonesia, and India	Mar–Nov 2020	12,915
King [42]	USA	Jan–May 2021	525,809
Kreps [43]	USA	Jul 2020	1971
Kwok [44]	Hong Kong	Mar–Apr 2020	1205
Latkin [45]	USA	Mar 2020–Nov 2020	592
Lin [46]	China	May 2020	3541
Liu [47]	USA	Jan–Mar 2021	443,680
Machida [48]	Japan	Jan–Apr 2021	2655
Mellis [49]	USA	Sep 2020	87
Mueangpoon [50]	Thailand	Sep 2021–Jan 2022	705
Orangi [51]	Kenya	Feb 2021	4136
Park [52]	South Korea	Feb 2021	1000
Parsons [53]	Canada	Feb–May 2021	60
Pogue [54]	USA	n.a.	316
Reiter [55]	USA	May 2020	2006
Riad [56]	Czech Republic	Apr–Jun 2021	1351
Rozek [57]	17 countries	15,151	
Rzymski [58]	Poland	Feb–Mar 2021	1020
Simione [59]	Italy	Apr 2020	350
Schernhammer [60]	Austria	Nov–Dec 2020	1007
Soares [61]	Portugal	Sep 2020–Jan 2021	1943
Stoler [62]	USA	Jun 2020	1040
Strupat [63]	Ethiopia	Nov 2020	2317
Szilagyi [64]	USA	Dec 2020–Jan 2021	5979
Trent [65]	USA, UK, Australia	Jul–Sep 2020	2712
Viswanath [66]	USA	Jul 2020	1012
Wang [67]	China	Jan 2021	8742
Williams [68]	Italy	Jan–Feb 2021	3893
Willis [69]	USA	Jul–Aug 2020	1205

**Table 2:** Typologies of components of trust that have been characterized as predictors of *vaccine acceptance* in the considered contributions.

	Positively Related	Negatively Related	Occurrences
**Trust in the COVID-19 vaccine**	Abdou [27], Dorman [32], Falcone [33], Gerretsen [35], Han [37], Jafar [39], Jennings [40], King [42], Liu [47], Machida [48], Mueangpoon [50], Parsons [53], Soares [61], Trent [65], Wang [67]		15
**Vaccine’s safety**	Babicki [29], Dorman [32], Gerretsen [35], Han [37], Kerekes [41], King [42], Kreps [43], Lin [46], Mellis [49], Mueangpoon [50], Orangi [51], Park [52], Parsons [53], Pogue [54], Reiter [55], Soares [61], Szilagyi [64], Trent [65], Wang [67], Williams [68]		20
**Vaccine’s efficacy**	Babicki [29], Fernández [34], Gerretsen [35], Goodwin [36], Han [37], Kerekes [41], Kreps [43], Lin [46], Mueangpoon [50], Orangi [51], Pogue [54], Reiter [55], Soares [61], Szilagyi [64], Trent [65], Wang [67], Williams [68]		17
**Concerns about commercial profiteering**		Gerretsen [35]	1
**General trust in vaccines**	Brindle [30], Kwok [44], Parsons [53], Willis [69]		4
**Trust in government**	Allington [28], Brindle [30], Gerretsen [35], Goodwin [36], Jennings [40], King [42], Mellis [49], Mueangpoon [50], Park [52], Parsons [53], Rozek [57], Schernhammer [60], Strupat [63], Szilagyi [64], Trent [65], Wang [67]	Trent [65]	16
**Trust in health system**	Allington [28], Jennings [40], Reiter [55], Riad [56], Rozek [57], Simione [59], Soares [61], Stoler [62], Szilagyi [64]		9
**Trust in sciences**	Allington [28], Brindle [30], Fernández [34], Jennings [40], Rozek [57], Simione [59], Viswanath [66]		7
**Trust in religion**		Rozek [57]	1
**Social trust**	Jennings [40]		1
**Trust in COVID-19 manufacturers**	Riad [56], Szilagyi [64], Wang [67], Williams [68]		4

**Table 3:** Contributions considering both COVID-19 vaccine acceptance and COVID-19 vaccine trust.

Contribution	Vaccine Acceptance	Trust in the COVID-19 Vaccine
Abdou [27], multi-country, December 2020–February 2021	27.9%	26.7%
Daly [31], USA, October 2020–March 2021	64.8%	50%
Falcone [33], Italy, March–April 2021	88.8%	92.9%
Han [37], Cina, November 2020	89.1%	82%
Lin [46], China, May 2020	83.3%	94.8%
Liu [47], USA, January–March 2021	84.1%	90%
Machina [48], Japan, January 2021–April 2021	72.4%	58.7%
Mueangpoon [50], Thailand, September 2021–January 2022	89.6%	60.6%
Soares [61], Portugal, September 2020–January 2021	35%	58%
Wang [67], China, January 2021	67.1%	69%

## Data Availability

Not applicable.

## Abbreviations

The following abbreviations are used in this manuscript:

CDC	Centers for Disease Control and Prevention
n.s.	not significant
SAGE	Strategic Advisory Group of Experts on Immunization
WHO	World Health Organization
